# Pedestrian Detection with Semantic Regions of Interest

**DOI:** 10.3390/s17112699

**Published:** 2017-11-22

**Authors:** Miao He, Haibo Luo, Zheng Chang, Bin Hui

**Affiliations:** 1 Shenyang Institute of Automation, Chinese Academy of Sciences, Shenyang 110016, China; luohb@sia.cn (H.L.); changzheng@sia.cn (Z.C.); huibin@sia.cn (B.H.); 2 Key Laboratory of Opto-Electronic Information Processing, Chinese Academy of Sciences, Shenyang 110016, China; 3 The Key Lab of Image Understanding and Computer Vision, Shenyang 110016, China; 4 University of Chinese Academy of Sciences, Beijing 110049, China

**Keywords:** pedestrian detection, deep learning, background vs. foreground errors, semantic regions of interest

## Abstract

For many pedestrian detectors, background vs. foreground errors heavily influence the detection quality. Our main contribution is to design semantic regions of interest that extract the foreground target roughly to reduce the background vs. foreground errors of detectors. First, we generate a pedestrian heat map from the input image with a full convolutional neural network trained on the Caltech Pedestrian Dataset. Next, semantic regions of interest are extracted from the heat map by morphological image processing. Finally, the semantic regions of interest divide the whole image into foreground and background to assist the decision-making of detectors. We test our approach on the Caltech Pedestrian Detection Benchmark. With the help of our semantic regions of interest, the effects of the detectors have varying degrees of improvement. The best one exceeds the state-of-the-art.

## 1. Introduction

Pedestrian detection is a canonical instance of object detection [1]. It is a challenging but important problem because it is a key technology in automotive safety, robotics and intelligent video surveillance. As these tasks have attracted much attention in the last few years, more and more researchers are involved in the study of pedestrian detection.

In response to the challenges of pedestrian detection, three methods are often mentioned by researchers: HOG (Histogram of Oriented Gradient) + SVM (Support Vector Machine) rigid templates, deformable part detectors (DPM) and convolutional neural networks (ConvNets) [1]. For a long time, pedestrian detection methods were basically based on HOG + SVM published in Conference on Computer Vision and Pattern Recognition (CVPR) 2005, worked out by French researchers Dalal and Triggs [2]. In recent years, deep learning has been the most popular method in the field of image processing. It has achieved perfect results in tracking, detection, segmentation and other fields.

In our research, it is found that many algorithms, such as the HOG + SVM, Aggregated Channel Features (ACF) [3] and JointDeep [4], detect many candidate boxes with no pedestrian and raise the error rates. Reducing background vs. foreground errors can raise the accuracy of these algorithms substantially. However, fully-convolutional networks trained end-to-end, pixel-to-pixel can distinguish the background and foreground effectively and reduce this kind of error.

In this paper, a two-category fully-convolutional neural network is trained with the Caltech Pedestrian Dataset to convert the original image into a heat map, which is sensitive to pedestrians. By using the technology of morphological image processing, we process the heat map into semantic regions of interest. With the regions of interest, HOG + SVM finds less false positives, and the miss rate is reduced from 69% to 54% on the Caltech Pedestrian Detection Benchmark. The frames detected per second is increased from 0.054 to 1.123. Furthermore, our semantic regions of interest can also work on many other algorithms. Their best result can exceed the state-of-the-art. In addition, our approach show good generalization capability on the TUD-Brussels Pedestrian Dataset and the ETH Pedestrian Dataset. It is shown that the semantic regions of interest have an auxiliary improving effect on pedestrian detectors.

The rest of the paper is organized as follows. In Section 2, we introduce the related research work about deep neural networks and pedestrian detection. In addition, we show the defect of existent algorithms here. In Section 3, we describe the detail of our approach about the label processing, the visual geometry group, the heat map, end-to-end training, semantic regions of interest and combining with other algorithms. The results of the experiments on the Caltech-USA pedestrian detection dataset and two additional datasets are presented in Section 4. Section 5 concludes the paper.

## 2. Related Work

Our approach draws on the success of deep neural networks for feature extraction. Feature extraction using deep neural networks can be traced back to LeNet [5]. Thanks to the ImageNet project, since 2012, more and more network structures have been developed for image classification tasks [6,7,8,9,10,11,12]. These network structures have made significant contributions to the field.

Because the training of deep neural networks requires a large amount of data and it takes such a long time to achieve convergence, transfer learning has become a necessary method. It was proven by Yosinski et al. that transferring features even from distant tasks could be better than using random initialization [13].

With the help of the new networks and transfer learning, deep learning has achieved great success in various high-level visual tasks. This enables researchers to address tasks that are more challenging, such as semantic segmentation. Semantic segmentation is a kind of pixel-level labeling problem. At present, the most popular semantic segmentation methods are basically based on the fully-convolutional network (FCN) by Long et al. [14]. In the FCN, well-known classification models, including AlexNet, VGG-16, GoogLeNet and ResNet, were transformed into a fully-convolution model to learn hierarchies of features. At the same time, the fully-connected layers were replaced by convolutional ones to output spatial maps. These maps were obtained by fractionally stridden convolutions (also known as deconvolution) to generate dense pixel level labels.

The most popular pedestrian detector is the sliding-window-based method of Viola and Jones [15]. Their method consists of two main components: feature extraction and the AdaBoost classier [16]. However, there are still many important methods for obtaining detection proposals, including gPbUCM (Globalized Probability of Boundary, Ultrametric Contour Map) [17], CPMC (Constrained Parametric Min-Cuts) [18], Endres2010 [19], MCG (Multiscale Combinatorial Grouping) [20] and SelectiveSearch [21], built upon some form of hierarchical segmentation [22]. At the same time, there are methods for getting detection proposals with 3D information provided by stereo images or a Lidar sensor. These methods can fuse the 2D detector and the 3D detector to boost object detection and recognition confidence [23], or they can estimate the presence of objects above the ground in the image for obtaining proposals quickly [24], or prune the amount of the proposals generated by the classical pyramidal sliding window [25], with 3D information to speed up the detectors.

In CVPR 2005, researchers from France, Navneet Dalal and Bill Triggs proposed the approach for pedestrian detection with HOG for feature extraction and a linear SVM as the classifier [2]. They also discovered through many tests that HOG + SVM is a kind of pedestrian detection method with a good balance of speed and effect. Later, many researchers proposed many improved pedestrian detection algorithms based on it. HOG + SVM has been considered as a milestone. Since then, more and more kinds of pedestrian detection algorithms have been developed.

In International Conference on Computer Vision (ICCV) 2013, JointDeep was developed to get a unified deep model for joint learning with convolutional neural networks [4]. The unified deep model jointly learns four components—feature extraction, deformation handling, occlusion handling and classification—for pedestrian detection in order to maximize their strengths through cooperation. This algorithm became the best-performing pedestrian detection approach on the largest Caltech benchmark dataset at that time.

In 2014, Dollár et al. found that finely-sampled pyramids might be obtained inexpensively by extrapolation from coarsely-sampled ones, which decreases computational costs substantially. Based on this, ACF [3] was developed. They use the normalized gradient magnitude, histogram of oriented gradients and LUV color for features, which is the same as ChnFtrs [26]. Then, they compute the features at octave-spaced scale intervals to approximate features on a finely-sampled pyramid. Finally, they use AdaBoost [16] as the classifier. This algorithm was improved to be as accurate as the state-of-the-art in that year [27].

Paisitkriangkrai et al. developed a new algorithm named SpatialPooling in 2016 [28]. Their new features are built based on low-level visual features, such as covariance descriptors [29] and LBP (Local Binary Pattern) [30], and spatial pooling [31]. Incorporating spatial pooling improves the translational invariance and thus the robustness of the detection process. To achieve the optimal log-average miss rate performance measure, they learn another set of weak learners’ coefficients whose aim is to improve the detection rate at the range of most practical importance. The algorithm reached the best-reported performance on the Caltech-USA pedestrian detection dataset in 2016.

For now, the result of LDCF++(Locally Decorrelated Channel Feature with contextual reasoning) has gained the best training practice on Caltech [27,32]. The algorithm focuses on the relationship between modeling capacity of the weak learners, dataset size and dataset properties, inspired by the success of large, data-hungry visual recognition models. The performance is on par with deep architectures, while using only HOG + LUV channels as features.

**Figure 1:**
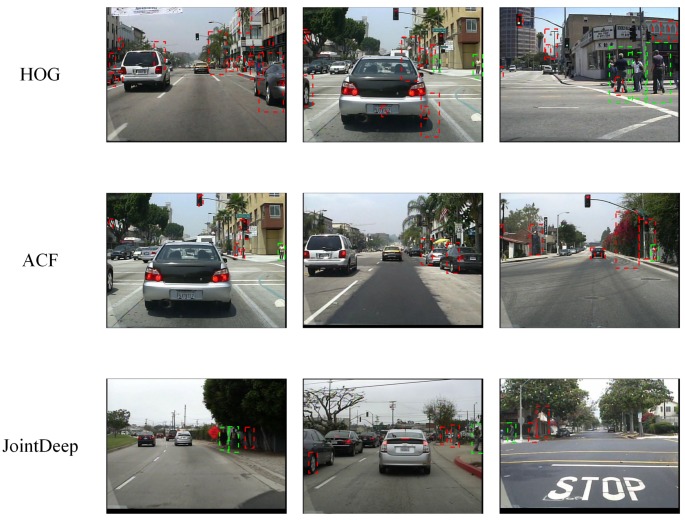
Many algorithms detect many candidate boxes with no pedestrian. Green boxes are true positives. Red ones are false positives.

In the research of Zhang et al. in 2016, it is found that for most methods, background vs. foreground errors have an important impact on the detection quality [33]. As shown in Figure 1, HOG + SVM, ACF and JointDeep detect many candidate boxes in the background with no pedestrian, which raise their error rates.

## 3. Our Approach

Our main idea is to reduce the background vs. foreground errors of the pedestrian detection algorithms by distinguishing the foreground from the background, so as to reduce the false positives per image. Semantic segmentation networks trained end-to-end, pixel-to-pixel can obtain the classification information of each pixel in the image. When the classification information is mapped to the original image, the foreground of the original image can be separated from the background area to assist the detection of the pedestrian detection algorithm and improve its effectiveness.

**Figure 2:**
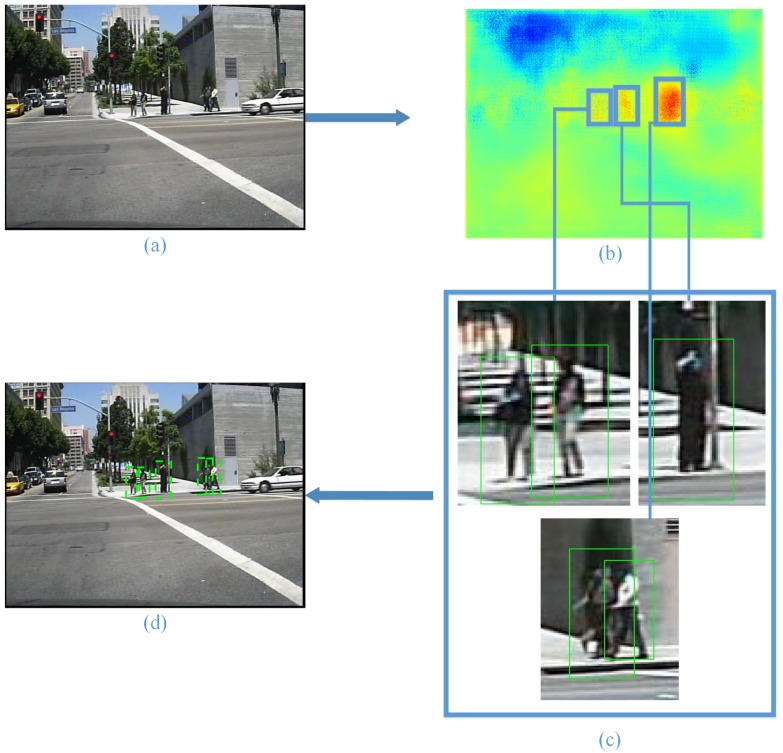
Key idea of our approach for pedestrian detection. (**a**) is the ordinary image, transferred to the heat map (**b**) with the deep neural network. The regions of interest are extracted from the heat map and then zoomed to the same scale. The HOG + SVM algorithm is used for pedestrian detection in the zoomed regions of interest (**c**). Finally, the detection results are mapped to the ordinary image (**d**).

First, a two-category fully-convolutional neural network is trained to output a heat map, which is sensitive to pedestrians. In order to obtain a better detection algorithm that does not rely on additional data, the fully-convolutional neural network was trained on a dataset for detection instead of one with pixel-level annotations. For this reason, the fully-convolutional neural network is introduced to extract regions of interest, instead of pedestrian contours. Next, with the help of morphological image processing, we get the regions of interest. Finally, we introduce HOG + SVM as an example to perform pedestrian detection and show the improvement; as shown in Figure 2.

For the HOG + SVM pedestrian detector, there is a sliding window moving on the whole image. To deal with the problem of multiple scales, an image pyramid with many layers is needed. However, in our approach, firstly, we obtain the regions of interest (ROI) in the image with semantic segmentation networks. Then, the sliding window moves on the ROI instead of the whole image, which reduces the false positives and raises the speed of the algorithm. At the same time, because of the accuracy of the ROI, we only need an image pyramid with a few layers to deal with some special situation, which raises the speed of the algorithm again.

### 3.1. Label Processing

To use a detection dataset in fully-convolutional neural networks, the labels need to be transformed. The labels are bounding boxes in the format “[picID, left, top, width, height]”. To adapt the labels to the fully convolutional neural networks, for each picture, we create a two-dimensional matrix with the same size and make the values of the pixels that are inside the bounding box one, otherwise zero.

**Figure 3:**
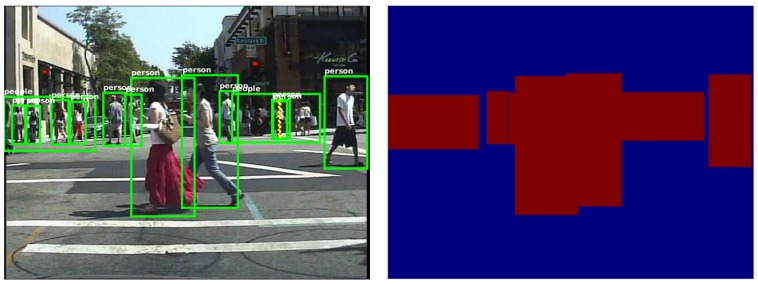
Labels of the dataset (**left**) and the transformed one (**right**). In the right picture, values in the red area are set to one, in the blue area to zero.

Labels in the dataset can be divided into two categories, people and person. For our task of extracting heat maps, since the pixels in both of the categories belong to the region with a human in it, we do not distinguish between the two categories. In the transformed label, all the pixels in these regions are set to be one. The labels before and after being transformed are shown in Figure 3.

### 3.2. Visual Geometry Group

**Figure 4:**
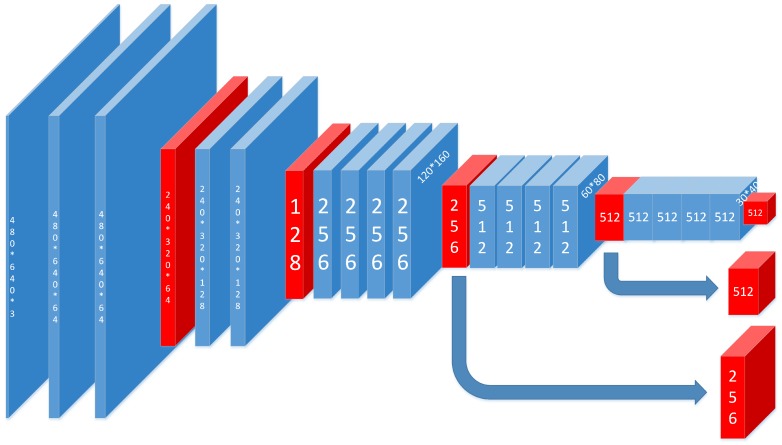
The figure maps of VGG19 in our approach. Blue ones are got from convolutional layer, and red ones are got from max-pooling layer. All the convolution kernels are in the size of 3 × 3. The size of the output is 15 × 20, 1/32 of the original image. At the same time, the figure maps got from pooling3 and pooling4 are also sent to next stage.

The visual geometry group (VGG) is introduced for feature extraction. In this paper, our feature extraction is conducted based on the 19-layer VGG network that has been pre-trained on the ImageNet image classification task. As shown in Figure 4, the feature map selecting method we proposed is based on the pooling3, pooling4 and pooling5. With these feature maps, features of different scales are extracted. For the original VGG, it was used to cope with classification tasks; the input picture should be square, and the resolution should be 224 × 224. However, our purpose is to get the heat map, and the input should be the ordinary image with the resolution being 480 × 640. For this reason, all the fully-connected layers are abandoned, and then, we can keep the resolution of our input and use the pre-trained model at the same time.

### 3.3. Heat Map

**Figure 5:**
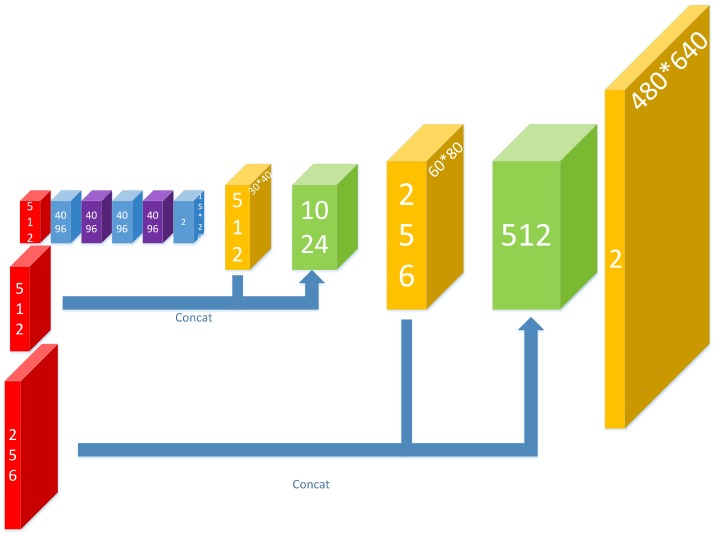
The maps of the heat map network in our approach. Red ones are obtained from the last stage, blue ones from convolutional layers, purple ones from dropout layers, yellow ones from deconvolutional layers and green ones from concat layers.

After the features have been extracted, a neural network to obtain the heat map of the regions of interest is constructed, as shown in Figure 5.

First, three convolutional layers are used to further abstract the features of pooling5. Two deconvolutional layers follow the layer of conv8 (the last convolutional layer), with the stride being two and the convolution kernel being 4 × 4.

The high-layer features are more abstract and better at capturing semantic concepts on object categories, but their receptive field is larger and less sensitive to location information, while the low-layer features’ receptive field is smaller and their location information more accurate [34]. In the task of getting the heat map, we should consider the accuracy of classification and location at the same time, which requires us to combine high-layer features with low-layer features. For this reason, the feature maps of the deconvolutional layers are concatenated with the feature maps of pooling4 and pooling3 respectively at the same time. With the help of the lower level feature map, the output results are more accurate in location.

At the end of the network, there is a deconvolutional layer with the stride being eight and the convolution kernel being 16 × 16, making the size of the output heat map the same as the ordinary image.

### 3.4. Details of Training End-To-End

In our approach, we use the Adam optimizer for training. The learning rate is set to 10${}^{-6}$, and the batch size is set to five. The forward propagation of the network outputs an array with the shape of (5480, 6402). The shape of the ground truth is (5480, 6401). Loss is set to reduce the mean of sparse softmax cross-entropy with logits of the output and ground truth. The softmax regression is defined as:

(1) $$p_{k}\left(x\right)=\frac{e^{a_{k}\left(x\right)}}{\sum_{k^{\prime }=1}^{K}e^{a_{k^{\prime }}\left(x\right)}}$$

where $a_{k}\left(x\right)$ denotes the activation in feature channel *k* at the pixel position x∈Ω with $\Omega \subset Z^{2}$. The softmax cross-entropy is defined as:

(2) $$L=\sum_{x\in \Omega }\log\left(p_{l_{x}}\left(x\right)\right)$$

where $l_{x}$ denotes the ground truth at the pixel position x∈Ω. The loss is set to be the average value of *L* at each pixel.

The weights are learned end-to-end by backpropagation from the pixel-wise loss. The whole dataset is trained for nine epochs, and the process takes three days.

### 3.5. Semantic Regions of Interest

Through the deep neural network, the heat map is obtained, and then, the graphics method is introduced to extract the semantic regions of interest (SROI) from the heat map.

**Figure 6:**
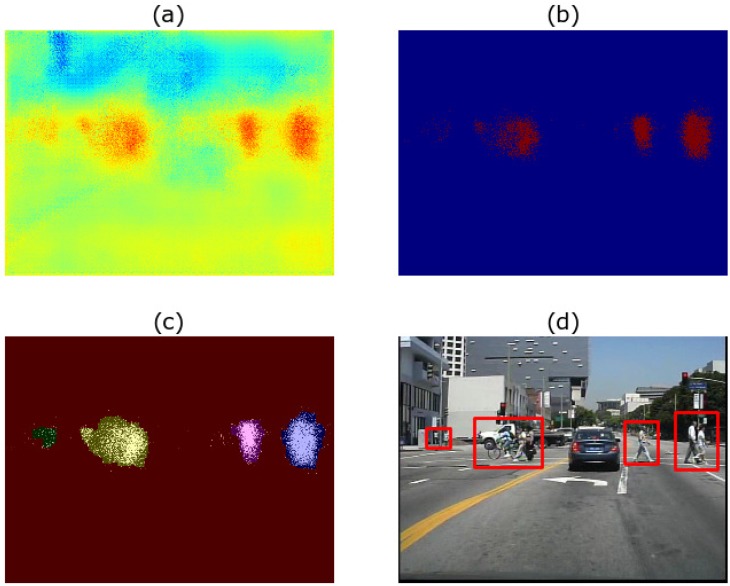
The process from the heat map to regions of interest. (**a**) is the heat map we obtained from the last stage. Then, it is transferred into a binary image (**b**). With the help of the morphology opening and closing operation, the noise is removed, and a series of connected regions is obtained at the same time in (**c**). Finally in (**d**), the bounding boxes of the connected regions are mapped to the original image to obtain the semantic regions of interest (SROI).

First, binary images are obtained from the heat maps with threshold segmentation. The values of the pixels in the heat map are limited between zero and one because of the softmax-layer, so the threshold was set to 0.4 to reduce the miss rate and raise the recall. Then, morphological closing and opening are operated to link the adjacent regions together to prevent repeated detections of the same target due to the pad of HOG. Next, small areas are filtered out, leaving only reliable areas. For all remaining areas, take their bounding boxes as the regions of interest. The whole process is shown in Figure 6.

### 3.6. HOG

First, we zoom the image of each region of interest to the same scale. With the help of deep learning, the division of the regions of interest is more accurate. The height of the SROI is basically the same as the height of the person in the region. This solves the problem that the HOG feature is not robust to scale variations. Because of this, we do use a HOG + SVM pedestrian detector combined with image pyramids to deal with the situation that people in different scales, such as an adult and a child, are in the same SROI, but the image pyramids did not need to have so many levels as usual. Besides, the SROI is much smaller than the image size. Therefore, the detection speed of HOG + SVM is greatly improved.

**Figure 7:**
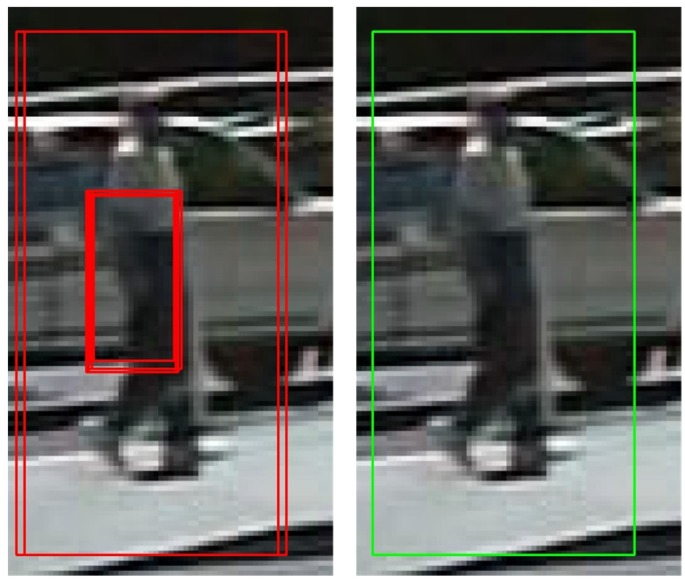
Results without non-maximum suppression (**left**) and with non-maximum suppression (**right**).

At the same time, because HOG + SVM pedestrian detection relies on the sliding window, we need to use non-maximum suppression to reduce redundant boxes, as shown in Figure 7.

## 4. Experiments

The proposed detector is implemented based on the wrapper of the TensorFlow framework and runs on a PC with a 3.5-GHz CPU and a TITAN X GPU.

In this session, we present the result achieved by our algorithm on the well-established Caltech Pedestrian Dataset. The pictures in the dataset were taken from a vehicle driving through regular traffic in an urban environment. The resolution of the pictures is 640 × 480. It is a large dataset with about 350,000 bounding boxes and 2300 unique pedestrians. The training data consist of six training sets; each set contains 6–13 files. In each file, there are about 1800 pictures with annotation information. The testing data consist of five sets with the same size.

Furthermore, we test our approach on another two datasets, the TUD-Brussels Pedestrian Dataset and the ETH Pedestrian Dataset, with the model trained on the Caltech Pedestrian Training Dataset to test the generalization capability. The TUD-Brussels Pedestrian Dataset is a dataset with image pairs recorded in a crowded urban setting with an onboard camera [35]. The ETH Pedestrian Dataset is an urban dataset captured from a stereo rig mounted on a stroller [36].

### 4.1. Results on Caltech Pedestrian Dataset

#### 4.1.1. Results Compared with Hog

**Figure 8:**
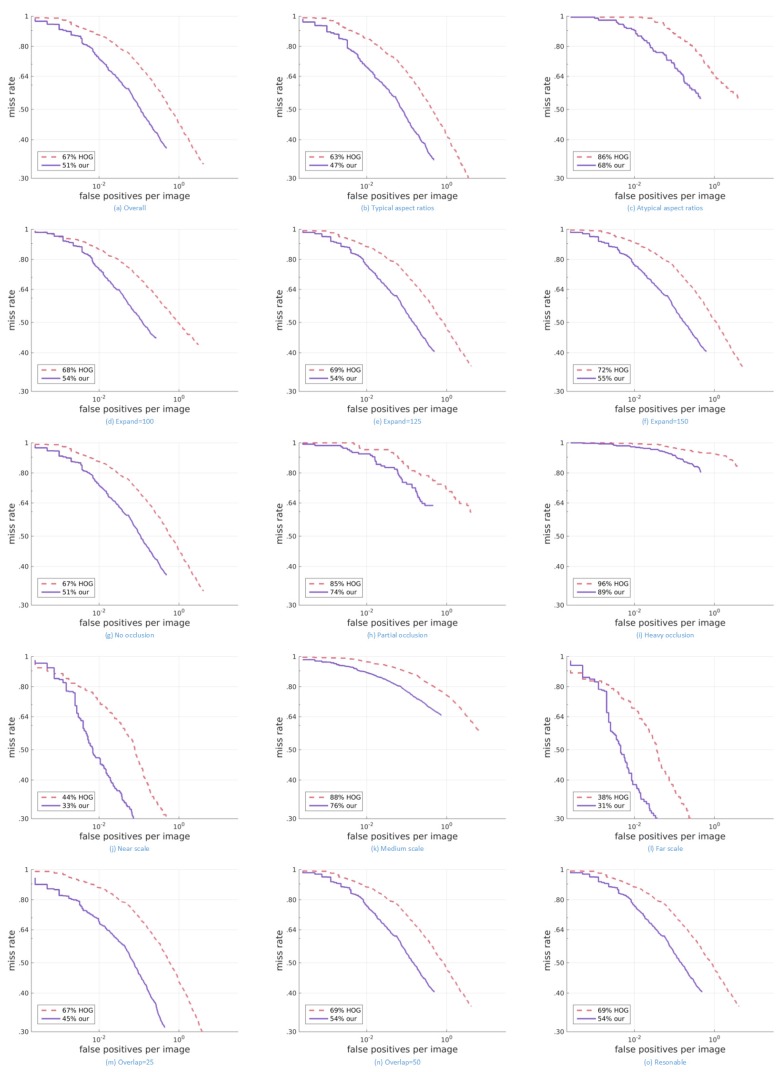
Result on the Caltech Pedestrian Detection Benchmark compared with HOG + SVM.

As shown in Figure 8, the results of our algorithm on various subsets of the dataset are better than those of HOG. Our algorithm can achieve almost the same miss rate with less false positives.

**Figure 9:**
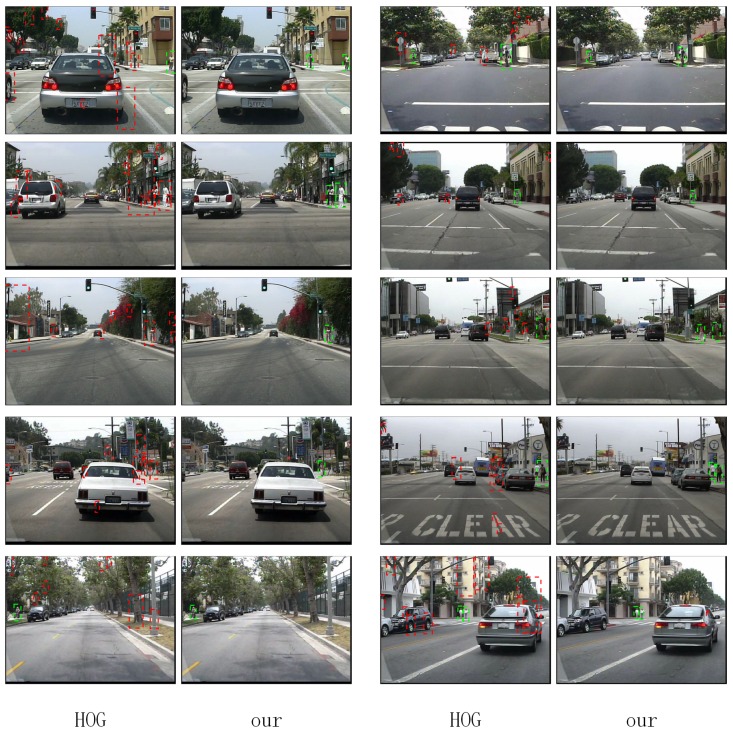
Results of HOG + SVM and our approach.

With our SROI, the miss rate of HOG + SVM is reduced from 69% to 54% on the reasonable subset of the Caltech Pedestrian Detection Benchmark. As shown in Figure 9, false positives in the background have been reduced effectively. At the same time, the speed of the detector has been effectively improved. The frames detected per second are increased from 0.054 to 1.123.

#### 4.1.2. Results of Other Algorithms with Our SROI

**Figure 10:**
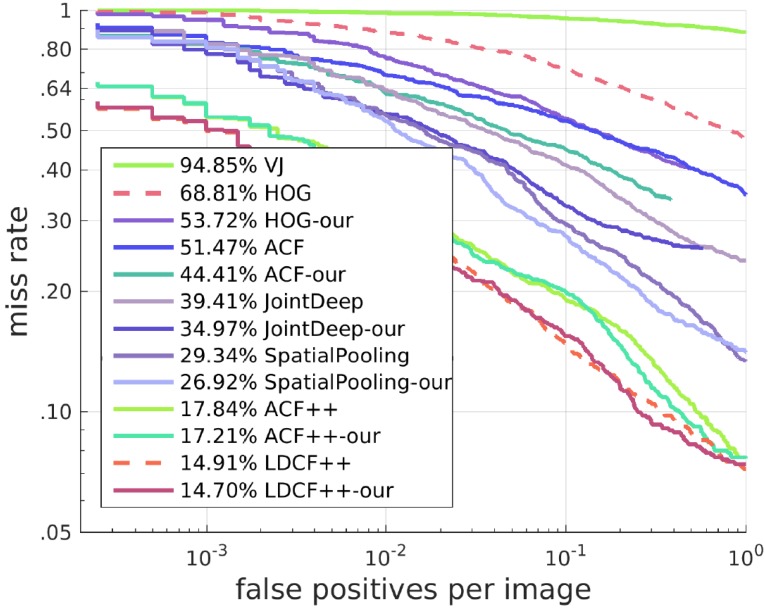
ROC curves of algorithms with and without our SROI.

**Figure 11:**
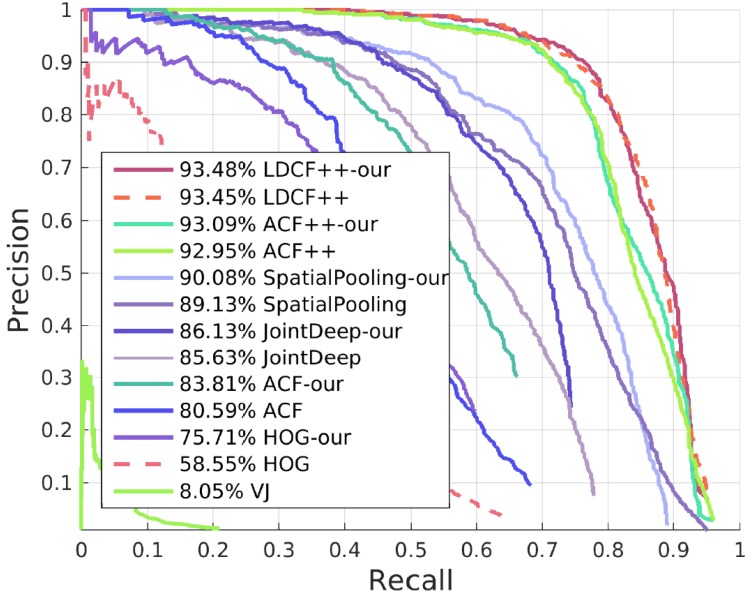
PR curves of algorithms with and without our SROI.

Our approach can also work on many other algorithms and solve similar problems to a certain degree. We deal with these algorithms with a different method from the one for HOG + SVM. Here, we keep all candidate boxes of these algorithms. When candidate boxes are in our SROI, their scores are increased proportionally, and when the candidate boxes are outside our SROI, their scores remain unchanged. This method has a good effect on improving the detector. The best result with our SROI can exceed the state-of-the-art. Receiver operating characteristic (ROC) curves and Precision-Recall(PR) curves of the results are shown in Figure 10 and Figure 11.

### 4.2. Results on the TUD-Brussels Pedestrian Dataset

**Figure 12:**
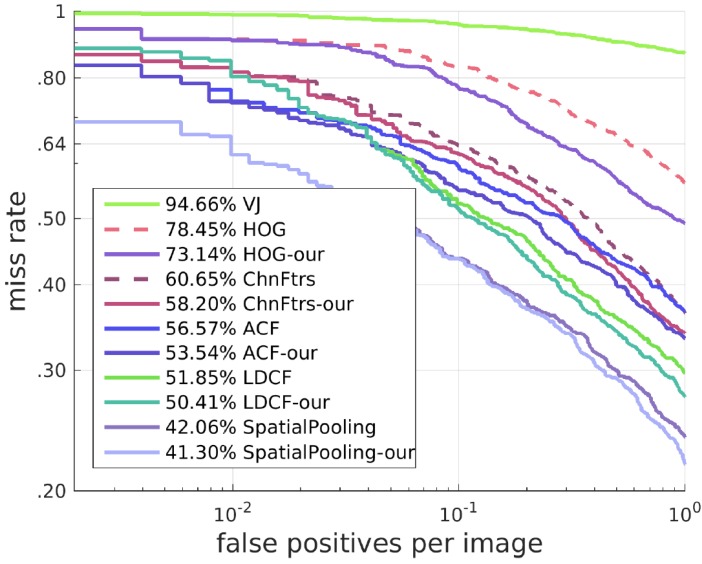
Results on the TUD-Brussels Pedestrian Dataset.

**Figure 13:**
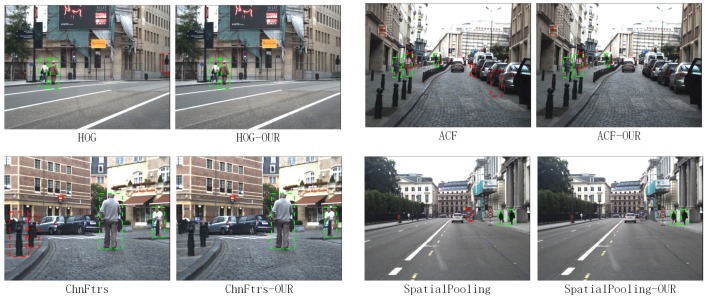
Results on the TUD-Brussels Pedestrian Dataset.

To test the generalization capability, we test our approach on the TUD-Brussels Pedestrian Dataset with the model trained on the Caltech Pedestrian Training Dataset without fine-tuning. With respect to the ROC curves shown in Figure 12, our algorithms is still effective on this dataset in improving the detectors. The detection results of different algorithms with our SROI on this dataset are shown in Figure 13.

### 4.3. Results on the ETH Pedestrian Dataset

**Figure 14:**
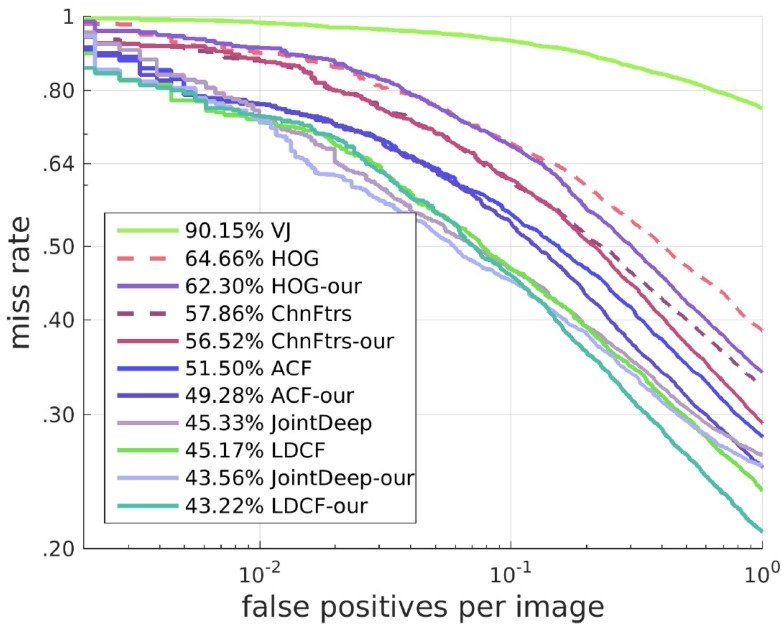
Results on the ETH Pedestrian Dataset.

**Figure 15:**
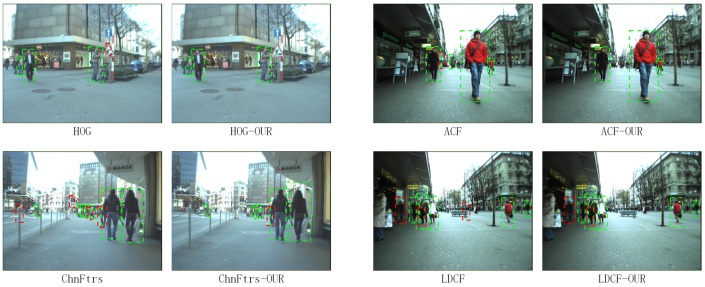
Results on the ETH Pedestrian Dataset.

Finally, we test our approach on the ETH Pedestrian Dataset, which is recorded from a different perspective from the previous two datasets. Instead of being captured from a vehicle in the middle of the road, this dataset is recorded from a stroller on the sidewalk. We test our approach with the model trained on the Caltech Pedestrian Training Dataset without fine-tuning. With respect to the ROC curves shown in Figure 14, our approach can still improve the performance of the detector. The detection results of different algorithms with our SROI on this dataset are shown in Figure 15.

## 5. Conclusions

In this paper, we improve the HOG + SVM pedestrian detector with our semantic regions of interest obtained from a fully-convolutional neural network. Trained end-to-end, pixel-to-pixel, fully-convolutional networks can distinguish the background and foreground effectively to reduce the background vs. foreground errors of the detectors. We test our SROI with HOG + SVM on the Caltech Pedestrian Dataset. Because the false positives are reduced substantially, the missing rate is reduced by about 15%. At the same time, the speed of the algorithm is improved effectively. Furthermore, we test our SROI with many other detectors, such as ACF, JointDeep, SpatialPooling and LDCF++, on the Caltech Pedestrian Detection Benchmark. Based on our experiments, we observe that the SROI can also work on these detectors in improving their accuracy. The best result with our SROI can exceed the state-of-the-art. In addition, our approach is tested on two other datasets and shows a good generalization capability.
